# Liver Transplantation for Hepatocellular Carcinoma beyond the Milan Criteria: A Specific Role for Living Donor Liver Transplantation after Neoadjuvant Therapy

**DOI:** 10.3390/cancers16050920

**Published:** 2024-02-25

**Authors:** Oliver Rohland, Lea Freye, Laura Schwenk, Aladdin Ali-Deeb, Michael Ardelt, Astrid Bauschke, Utz Settmacher, Falk Rauchfuß, Felix Dondorf

**Affiliations:** 1 Department of General, Visceral and Vascular Surgery, Jena University Hospital, 07747 Jena, Germany; 2 Interdisciplinary Center for Clinical Research (IZKF), Jena University Hospital, 07747 Jena, Germany; 3 Cancer Center Central Germany (CCCG), 04103 Leipzig, Germany

**Keywords:** hepatocellular carcinoma, Milan criteria, living donor liver transplantation, transplant outcome

## Abstract

**Simple Summary:**

This research delves into better treatment options for patients with liver cancer (HCC) who fall outside the eligibility criteria of traditional Milan guidelines for liver transplants. By reviewing patient data from Jena University spanning from 2007 to 2023, the study explores whether new patient classification systems and the use of living donor liver transplantation (LDLT) could extend life-saving options to those previously considered unsuitable. Findings indicate that patients not meeting the Milan criteria still benefit significantly from transplantation, showcasing similar survival rates between those undergoing standard transplants and LDLT. Key factors such as tumour grade and vascular invasion emerged as predictors for cancer recurrence, highlighting the importance of pre-transplant treatments in enhancing survival outcomes. The study underscores LDLT as a feasible alternative, particularly for patients undergoing successful bridging therapies, thereby broadening the scope of liver transplantation for liver cancer and offering new therapeutic approaches for advanced liver tumours.

**Abstract:**

Purpose: This study was designed to elucidate the various new classifications and the use of LDLT and bridging therapy for HCC in this context beyond the Milan criteria (MC). Methods: The clinical data of patients with HCC outside the MC who underwent LT at Jena University between January 2007 and August 2023 were retrospectively analysed. Eligible patients were classified according to various classification systems. Clinicopathological features, overall and disease-free survival rates were compared between LT and LDLT within the context of bridging therapy. The Results: Among the 245 patients analysed, 120 patients did not meet the MC, and 125 patients met the MC. Moreover, there were comparable overall survival rates between patients outside the MC for LT versus LDLT (OS 44.3 months vs. 28.3 months; 5-year survival, 56.4% vs. 40%; *p* = 0.84). G3 tumour differentiation, the presence of angioinvasion and lack of bridging were statistically significant risk factors for tumour recurrence according to univariate and multivariate analyses (HR 6.34; *p* = 0.0002; HR 8.21; *p* < 0.0001; HR 7.50; *p* = 0.0001). Bridging therapy before transplantation provided a significant survival advantage regardless of the transplant procedure (OS: *p* = 0.008; DFS: *p* < 0.001). Conclusions: Patients with HCC outside the MC who underwent LT or LDLT had worse outcomes compared to those of patients who met the MC but still had a survival advantage compared to patients without transplantation. Nevertheless, such patients remain disadvantaged on the waiting list, which is why LDLT represents a safe alternative to LT and should be considered in bridged HCC patients because of differences in tumour differentiation, size and tumour marker dynamics.

## 1. Introduction

Liver transplantation (LT) is so far the only curative option for patients with hepatocellular carcinoma (HCC) and accompanying liver cirrhosis [1]. HCC represents the most common malignant indication for LT [2].

The Milan criteria [3] (MC) were introduced in 1996 to assess the prognosis after LT for patients with hepatocellular carcinoma and liver cirrhosis and are still used today for organ allocation, with the standard exception of the MELD score, to prioritize patients with HCC inside the MC in the Eurotransplant area. In this region, organ allocation is based on the MELD system, and patients with hepatocellular carcinoma fulfilling the MC can generate exceptional points regardless of their labMELD score on the waiting list.

After more than 25 years of MC use, adjustments to the organ allocation criteria for patients with HCC outside of the Milan population are now being discussed, and even Mazzaferro et al. discussed expanding their own criteria over time [4].

Bridging procedures such as transcatheter arterial chemoembolization and selective internal radiotherapy have been able to further improve oncological outcomes in patients with HCC, and new therapeutic approaches such as immunotherapy have been established [5,6,7,8,9,10]. Even if immune checkpoint inhibition is currently not implemented in the bridging of patients with HCC on the waiting list, it is encouraging that this approach has the potential to further improve bridging therapy in combination with conventional bridging methods [8,11,12,13].

The present guidelines for HCC outside the MC are currently being further adapted internationally, for example, in the University of California San Francisco criteria (UCSF) [14], the extended Toronto criteria for liver transplantation [15], the up-to-seven rule [4], the Japanese 5-5-500 rule [16], adapted treatment recommendations for patients with hepatocellular carcinoma in Spain (Barcelona Clinic Liver Cancer classification) [17] and in the transplant regions of Australia and New Zealand [18].

The prognostic factors relevant for HCC in the context of LT were tumour morphology, tumour biology, tumour grade, cancer-related symptoms, the dynamics of the tumour biomarker alpha-fetoprotein, the response to bridging therapy [19] and the presence of angioinvasion.

Because the organ donation shortage in some transplant regions causes the waiting time and the average MELD score at the time of LT to continually increase, the probability of LT for patients with HCC outside the MC decreases, and thus, the number of HCC-associated deaths on the waiting list increases [20,21,22]. Therefore, the allocation criteria for these patients should be adjusted to allow them fair access to donor organs. The importance of the various new classification systems has not been conclusively assessed. These developments have led to the importance of LDLT for HCC patients, and an increasing LDLT is also being seen in non-Asian transplant centres [21].

The aim of the work was to show the results of liver transplants in patients with HCC inside and outside the MC and to compare the different HCC classifications with each other based on the results of the study population. Therefore, we wanted to demonstrate our bridging and transplantation strategies for these patients and the use of living liver donation. Overall survival (OS) and disease-free survival (DFS) were selected as the primary endpoints of this study. Markers for poor transplant outcome and oncological outcome were identified in our study population through univariate and multivariate analyses.

## 2. Materials and Methods

### 2.1. Patient Selection

The clinical data of the patients were collected through a retrospective review of medical records. Eligible patients were those who were diagnosed with HCC or who underwent LT for HCC between January 2007 and August 2023. Patient data were retrieved from the hospital database. Patients who underwent LT before January 2007 (before the introduction of the MELD score [23] system for organ allocation in the Eurotransplant area) were not included.

Details on organ allocation and information about the donor were obtained from the 245 patients via the Eurotransplant database (ENIS-next).

In addition, patients who were delisted from the waiting list were evaluated via the centre list during the period examined.

The response to bridging therapy was assessed by using the modified RECIST criteria for HCC and involved imaging every 3 months after bridging [24]. Each patient’s condition was discussed at an interdisciplinary tumour conference and also a transplantation conference [25].

### 2.2. Outcome Measures

All eligible patients who underwent LT or LDLT were stratified into two groups according to the MC. In the outside-Milan group, information about tumour location, bridging therapy, treatment response to bridging therapy, postoperative survival time, postoperative complications, hospital stay, ICU stay, tumour size, number of tumour nodes, histopathological findings, duration of alpha-fetoprotein perioperatively, and the timing of recurrence and treatment of the recurrence were investigated. The patients in the outside-Milan group were then further classified according to the expanded criteria (UCSF, Toronto, 5-5-500-rule, UTSC, BCLC).

Patients with HCC according to the MC were then categorized into two groups to compare the outcomes according to the surgical procedure (LDLT vs. LT).

Comparisons of preoperative factors, surgery-related factors, pathological findings, postoperative course, overall survival (OS) and disease-free survival (DFS) rates were performed between the LT and LDLT groups. The independently associated factors for OS and DFS were investigated using univariate and multivariate analyses.

### 2.3. Statistical Analysis

The normality test failed to confirm the data’s normal distribution. As a result, median values were determined, and statistical analysis proceeded with nonparametric methods (the Shapiro-Wilk test and the Kolmogorov-Smirnov test). Comparisons of clinical factors utilized the chi-squared test, Fisher’s exact test, and the Mann–Whitney U test. Disease-free survival (DFS) was determined from the transplantation date until the initial imaging-confirmed recurrence. Disease-free survival (DFS) and overall survival (OS) curves were generated through the Kaplan–Meier approach and assessed with log-rank tests. Hazard ratios (HRs) for DFS and OS risk factors, along with their 95% confidence intervals (CIs), were derived using the Cox proportional hazards model. Only factors deemed significant in the univariate analysis were considered for further multivariate analysis.

All the statistical analyses were performed using IBM^®^ SPSS Statistics 29 (IBM^®^, Armonk, NY, USA). A *p* value of <0.05 was considered indicative of statistical significance.

General medical data and parameters related to transplant outcomes were examined for the included patients. The patients were then grouped according to whether bridging, which transplant procedure was used (LT vs. LDLT) or whether they developed a recurrence.

## 3. Results

### 3.1. Epidemiology

**Figure 1:**
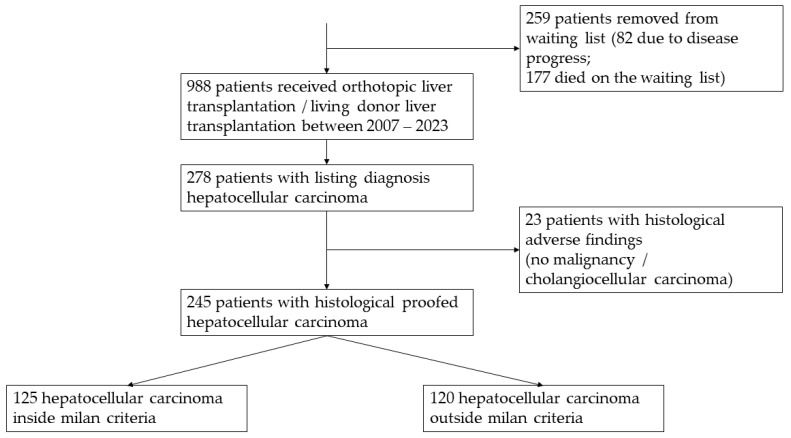
Selection progress.

A total of 245 patients who underwent LT for hepatocellular carcinoma at the Jena University Hospital Transplant Centre between January 2007 and August 2023 were enrolled in this study as shown in Figure 1. Of these, 125 (51%) patients met the MC, and 120 (49%) were outside the MC. The general epidemiological data for patients inside and outside the MC are presented in Table 1 and Table 2. LDLT was performed in significantly younger patients with lower MELD scores and larger tumours; these patients were less likely to be able to undergo bridging therapy successfully and were less likely to have decompensated liver cirrhosis. LDLT was always used when the urgency of the transplant in relation to the oncological outcome was not adequately reflected by the MELD system, and the time window for a promising transplant was therefore too short. As shown in Figure 1, many patients with HCC cannot receive transplantation in a timely manner and therefore have to leave the transplant waiting list due to disease progression and associated death.

The median follow-up period for all patients was 114 months.

### 3.2. Overall Survival Rate Inside and Outside the MC

In the group receiving LDLT within the MC, the median overall OS duration was 84.2 months, with 1- and 5-year survival rates of 100% and 78.8%, respectively (Table 1). For the group undergoing LT within the MC, the median OS spanned 62.8 months, with 1- and 5-year OS percentages at 99.1% and 80.1%, respectively. The comparison between the LDLT and LT groups did not reveal any statistically significant disparities (*p* = 0.30).

The median OS time in the LDLT outside-MC subgroup was 28.3 months, and the overall 1- and 5-year survival rates were 84.2% and 40%, respectively. The median OS time in the LT outside-MC group was 44.3 months, and the overall 1- and 5-year OS rates were 75.9% and 56.4%, respectively. OS was not significantly shorter in the LDLT group (*p =* 0.52).

### 3.3. Disease-Free Survival Rate Outside the MC and Factors Related to Disease-Free Survival Outside the MC

In the LDLT group, the median DFS was 32.2 months, with 1- and 5-year DFS rates at 76.3% and 56.5%, respectively (Table 2). In comparison, the LT group reported a median DFS of 62.3 months, with 1- and 5-year DFS rates of 85.7% and 72.3%, respectively. The LT group demonstrated significantly superior DFS compared to the LDLT group (*p* = 0.006).

Table 3 displays the univariate and multivariate analysis results for the risk factors affecting DFS. Tumour differentiation (G3), microvascular invasion and missing bridging therapy were significant risk factors for poor DFS in the univariate analysis (HR 6.34; *p* = 0.0002; HR 8.21; *p* < 0.0001; HR 7.50; *p* = 0.0001).

### 3.4. DFS and OS for Patients Who Underwent LDLT or LT Outside the MC

**Figure 2:**
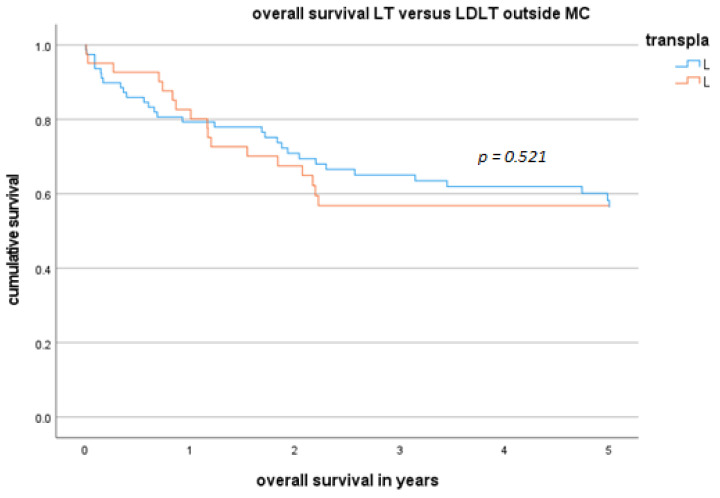

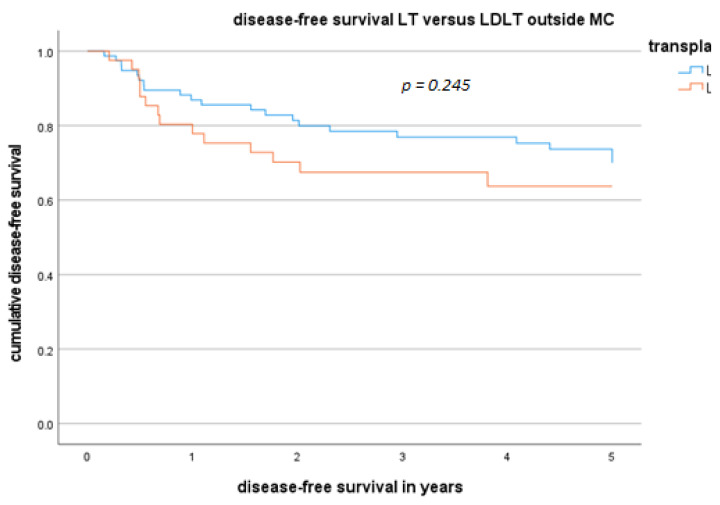
OS and DFS for LT versus LDLT outside the MC.

**Figure 3:**
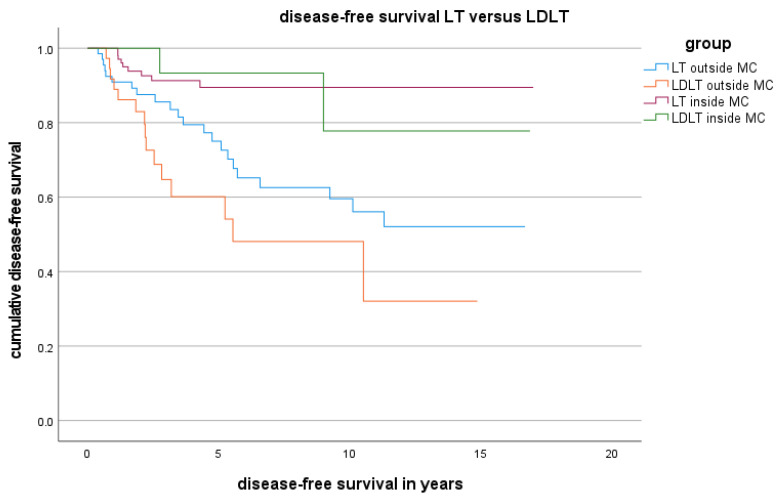
DFS for LT versus LDLT inside and outside the MC.

The DFS and OS of patients in the LDLT group and LT group are presented as Kaplan-Meier curves in Figure 2 and Figure 3. Although the OS in the LDLT group was lower, the groups did not significantly differ.

### 3.5. MELD Score, Tumour Morphology and Bridging Response Outside the MC

The median labMELD score was 10 in the LDLT group (mean 11.8; range 6–31) and 12 in the LT group (mean 16.7; range 6–40). The MELD score was significantly lower in the LDLT group (*p =* 0.005).

The median tumour size (determined by pathological examination) was 52 mm (mean 60 mm; range 30–220 mm) in the LDLT group and 48 mm (mean 48.4 mm; range 17–120 mm) in the LT group (*p* = 0.07).

The number of tumour nodes (determined by pathological examination) was 3 (mean 4; range 1–20) in the LDLT group and 2.5 (mean 4.2; range 1–25) in the LT group (*p =* 0.84).

**Figure 4:**
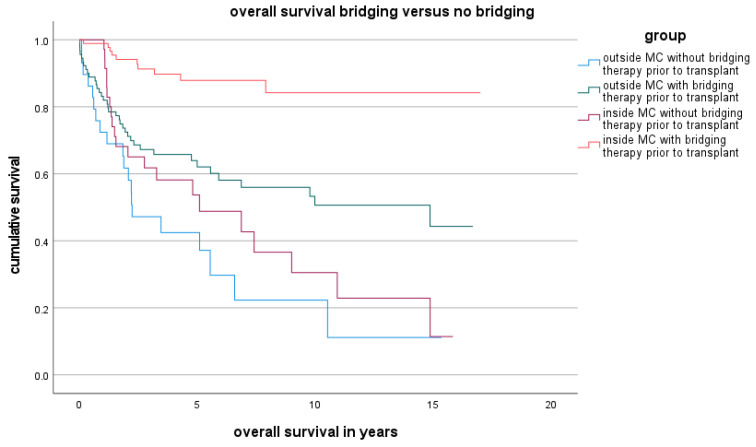
OS depending on bridging therapy inside and outside the MC.

**Figure 5:**
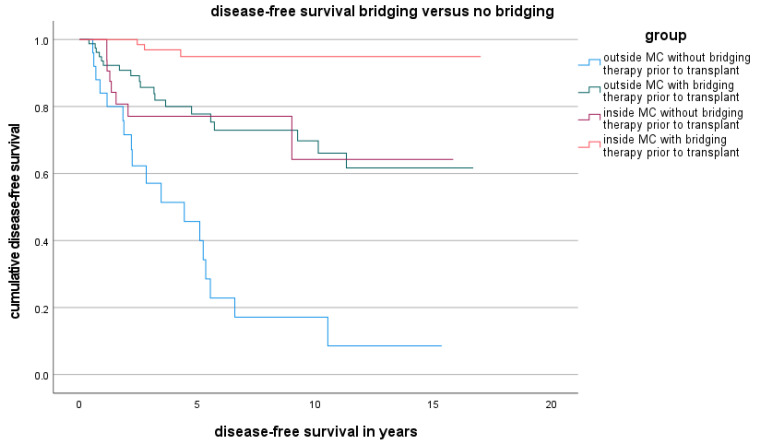
DFS depending on bridging therapy inside and outside the MC.

**Figure 6:**
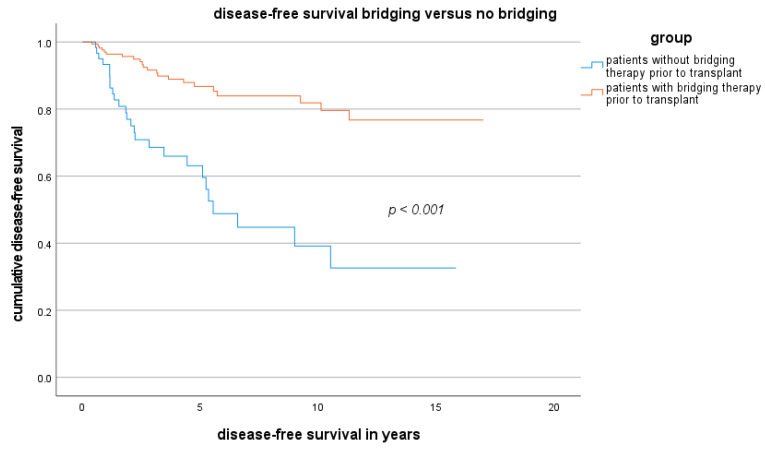
DFS was also stratified according to bridging therapy.

Downstaging after bridging therapy (regardless of the bridging method and frequency) was achieved for 43.8% of patients in the LDLT group and 50.7% of patients in the LT group (*p* = 0.52). A partial response after bridging therapy was observed in 59.4% of the LDLT group and 70.4% of the LT group (*p* = 0.27). Vital tumour residue after bridging was present in 18.8% of the LDLT group and 29.6% of the LT group (*p* = 0.25). The results for OS and DFS depending on bridging therapy were shown in Figure 4, Figure 5 and Figure 6.

### 3.6. Results According to Different Classifications beyond the Milan Grade

The classification results of the patients according to the different classifications (Milan, UCSF, 5-5-500, UPTS, Toronto and BCLC) are presented in Table 4. Our data showed that all the classifications used enabled the identification of patients who had a higher risk of developing recurrence after transplantation. However, it appears that the recurrence rate did not correlate with overall survival, disease-free survival, or 1-year or 5-year survival in any subgroup. The only classifications that were separated here were BCLC and Toronto. All (newer) classifications were deemed inferior to the MC according to the criteria above (survival rate and recurrence rate).

### 3.7. HCC Waitlist Dynamics

In addition to the transplanted patients, we also observed those who were diagnosed with HCC at our transplant centre. A total of 259 patients were removed from the waiting list during the observation period; 82 patients were no longer eligible for transplantation due to disease progression, and 177 died on the waiting list due to disease progression and liver failure.

## 4. Discussion

Liver transplantation is a curative procedure for the treatment of HCC inside the MC, but it also yields good but not comparable results for patients outside the MC and is superior to systemic therapy (OS 58.3 months versus 7.9–19.2 months) [26]. However, this treatment option is limited for patients due to organ shortages. LDLT can therefore be used for selected patients to enable those patients to undergo a transplant. Patients were selected on the basis of tumour biology markers such as AFP levels [27,28,29], tumour differentiation [30,31], response to bridging therapy [5,9,19] and the presence of cancer-related symptoms [15].

LT for patients with HCC outside the MC is still more demanding than for those with HCC inside the MC, but may constitute an established procedure with acceptable survival and recurrence rates at selected transplant centres because this procedure has been applied due to improvements in bridging therapy and patient selection, far from purely morphological aspects. Nevertheless, doubts remain about the equivalence of patients outside the MC; equivalence with patients inside Milan should be established according to the new extended criteria if bridging or even downstaging is possible.

Patients with hepatocellular carcinoma outside the MC have a significantly worse outcome than patients with HCC inside the MC if bridging therapy is not feasible. However, there are significant differences among the patients in the outside-Milan group, so that they should not be disadvantaged across the board in organ allocation.

LDLT is a good option for transplant patients with HCC who have no chance of receiving a liver transplant via a postmortem organ. We must clarify that the groups of patients who underwent LT or LDLT represented two different populations due to preselection as shown in the epidemiological distribution. Living donation should be considered, especially for young patients with low labMELD scores or poorly bridgeable or difficult-bridgeable HCC due to large or multifocal tumours.

The LDLT-patient group had a slightly worse outcome than the LT-patient group did, but this can be explained by selection bias since the factors mentioned above played an important role in patient selection in the LDLT group as shown in Table 2 (due to larger tumours, 52 mm vs. 48 mm, *p* = 0.07; higher number of tumours, 3 versus 2.5, *p* = 0.84; poorer bridging, 66.7% versus 79.7%, *p* = 0.12; poorer bridging response, 53.8% versus 70.4%, *p* = 0.24; poorer tumour differentiation, *p* = 0.01).

It is still controversial whether LDLT or LT is more beneficial for patients with HCC. A well-designed, randomized, controlled trial is needed, which is not possible. The existing studies addressing this topic have shown conflicting results [30,32,33,34,35,36,37].

Over the years, various additions to the MC have been made to push its boundaries. Starting with UCSF expansion to the MC, the first attempt was to adjust the tumour morphology, particularly the allowable tumour size, via a similar approach to that of Mazzaferro et al. [3,4].

Among the classifications considered to be related to morphological aspects beyond Milan, the UCSF classification expands the tumour size range (<80 mm) [14] compared to that of the MC. In our centre, we recommend a progressive strategy and perform transplants (via LDLT) regardless of tumour size because we consider other aspects, such as the response to bridging therapy, the dynamics of tumour markers and tumour grading, to be more relevant.

Only the extended Toronto criteria were used for other aspects, in addition to the tumour size and number of tumour nodules, which can predict a poor outcome. These criteria stand out among those included because they have no upper limit on size or number of lesions but exclude patients with cancer-related symptoms (weight loss >10 lbs or worsening performance status over 3 months) [15,38]. We support the basic idea of these criteria, but obligatory tumour biopsy for assessing microscopic angioinvasion needs to be discussed. There are no observations as to whether it makes sense to take the risk of carrying tumour cells during biopsy to detect the V1 situation. For this reason, we do not currently favour this approach.

The BCLC has been published as a guide for therapeutic decision-making in patients with HCC. Two new aspects, the general conditions and the liver parenchyma changes of the patients, were highlighted and influenced the outcome [17]. For this purpose, the classification system uses the Eastern Cooperative Oncology Group (ECOG) scale as a simple but effective diagnostic tool [39]. However, we consider the classification to be too regressive and would like to better demonstrate the role of living liver donation in the decision-making process.

As a further development of its own criteria, Mazzaferro’s working group has also published an adaptation of the MC for patients outside Milan [4]. In studies, the up-to-seven criteria showed no differences in MC [40]. We can share this assessment and consider the up-to-seven rule and MC to no longer be up to date [41].

In recent years, Asian transplant centres have also been working on further developments for prognosis assessments [14,16,42,43]. Among the various classifications, we have included the “5-5-500” rule here, which has gained increased attention due to its simplicity. Compared to the other classifications, this rule is relatively conservative regarding selection, and although it includes AFP as a tumour marker, it is not very revolutionary regarding morphological aspects. The use of the AFP concentration as a guide for predicting the outcome of HCC after LT has also been examined in various ways [19,27,29,31,44,45,46,47,48]. Our own experience shows that the preoperative AFP level alone determines oncological outcome in terms of DFS and OS. There is no clear cutoff for the AFP value for assessing the outcome of a transplant. Methodologically, the problem is that there is no clearly defined period during which the AFP level is meaningful, and the dynamics of the AFP level have thus far had no influence on the evaluation. In unbridged or inadequately bridged HCC, rapid AFP dynamics before transplantation can indicate a poor oncological outcome. We also observed that an insufficient or delayed decrease in the AFP value after liver transplantation can indicate early recurrence.

There have been further adjustments to the classification for HCC, which have not been discussed further here [28,43,49,50]. These include, for example, the modification of the TNM criteria for HCC from Pittsburgh [51] and the Hangzhou criteria [49] for LT at HCC. We have not included the Pittsburgh modified TNM criteria because the disadvantage of these TNM adjustments is the limited accuracy of the pre-transplant predictions of pTNM51.

The TNM classification of HCC has already been controversially discussed [28,43,51,52]. Our data show large discrepancies in tumour sizes and the number of tumour foci according to imaging and pathology. This restriction should ultimately be extended to all classifications with a purely morphological consideration of HCC.

In relation to bridging and recurrence, our data show that bridging should always occur if this approach is technically feasible to improve the OS and DFS of these patients. In the case of living liver donation, bridging should take place before transplantation whenever technically possible, even if the transplant is delayed.

Furthermore, our data showed that the occurrence of tumour recurrence is not necessarily associated with a poorer survival prognosis. On the one hand, this difference may be related to the improvement in relapse therapy efficacy, although not much has changed in terms of medication. In our patient population, a large proportion of patients with HCC recurrence after LT underwent surgery. This subgroup also exhibited improved overall survival compared with patients who were treated only with medication during relapse. We therefore recommend considering surgical therapy, depending on the patient’s condition, even in patients with oligometastasis.

Several limitations accompany this study, with the foremost being its retrospective, single-centre design, which inherently carries the risk of unanticipated biases that cannot be entirely eliminated. The patient cohort was limited in size and not comparable to that of Asian high-volume transplant centres; therefore, the importance of the findings is limited to Western centres. A Germany-wide or Eurotransplant-wide evaluation would certainly be useful here. Another point that arises from this is that we would like to submit a plea for adjustments to the organ allocation guidelines in the Eurotransplant region to address the disadvantage of patients with HCC outside Milan compared to patients without HCC on the waiting list. A suggestion for the Eurotransplant region would be to distribute SE-MELD points for patients with HCC outside the Milan range as well. However, this distribution should occur in a reduced form. Currently, patients with HCC inside the Milan range start with a matchMELD score of 22 points, and 3 additional points are added every 3 months if the MC are still met. The model for patients with HCC outside the Milan range could start with a reduced number of SE-MELD points and include a smaller gain to give these patients a realistic chance of a liver transplant.

## 5. Conclusions

Liver transplantation stands as the optimal treatment choice for patients diagnosed with HCC complicated with cirrhosis. LT for patients with HCC outside the Milan range should be considered regardless of the morphological aspects, for well- to moderately differentiated tumours (G1 or G2 differentiation), without microscopic angioinvasion and with favourable AFP dynamics if preoperative bridging therapy can be performed. Prompt LDLT after bridging therapy for HCC outside MC may be a solution in individual cases. The evidence from our study underscores the pivotal importance of bridging therapy for HCC cases outside MC, suggesting that advancements in these treatments are essential for improving transplant outcomes further.

## Figures and Tables

**Table 1:** Characteristics of patients with HCC inside the Milan range stratified by transplantation.

	LT (n = 106)	LDLT (n = 19)	*p* Value
Preoperative factors			
Age (median/range)	62 (40–70)	57 (23–69)	0.0001
Sex (male/female)	90/16	18/1	0.25
Cirrhosis (CPT A/B/C), n	27/66/10	6/7/5	0.73
No cirrhosis	2	2	0.05
Laboratory data (median, range)			
labMELD	11 (6–40)	16 (6–24)	0.58
SE-MELD	28 (22–39)	25 (22–31)	0.26
Preoperative AFP (mg/dL)	11.3 (1.0–1240)	12.9 (1.7–5675)	0.82
Bridging therapy (%)	67.9	47.4	0.22
Partial response and downstaging (%)	76.5	68.8	0.72
Vital tumour residue after bridging (%)	15.4	13.1	0.67
Pathological findings (n)			
Histological type (GX/G1/G2/G3)	54/29/21/2	10/5/3/1	0.96
Lymphatic permeation	2	1	0.38
Microvascular invasion	9	3	0.32
Perineural invasion	4	1	0.76
UICC T1/T2/T3/T4	50/42/14/0	8/8/3/0	0.66
UICC N0/N1/N2	106/0/0	19/0/0	
Postoperative course			
Overall survival in d	1882	2526	0.30
1 year survival (%)	99.1	100	0.67
5 year survival (%)	80.1	78.8	0.74

CPT, Child-Pugh-Turcotte Score for cirrhosis mortality; AFP, alpha-fetoprotein; GX, grade cannot be assessed due to neoadjuvant therapy; UICC, Union for International Cancer Control.

**Table 2:** Characteristics of patients with HCC outside the Milan range stratified by transplantation.

	LT (n = 79)	LDLT (n = 39)	*p* Value
Preoperative factors			
Age (median/range)	63 (44–71)	59 (23–71)	<0.0001
Sex (male/female)	69/10	34/5	0.98
Cirrhosis (CPT A/B/C), n	28/32/19	22/14/3	0.0001
Laboratory data (median, range)			
labMELD	12 (6–40)	10 (6–31)	0.005
Preoperative AFP (mg/dL)	14.45 (2.3–161974)	15.4 (1.9–28284)	0.93
Bridging therapy (%)	79.7	66.7	0.12
Partial response and downstaging (%)	70.4	53.8	0.24
Vital tumour residue after bridging (%)	29.6	18.8	0.25
Pathological findings (n)			
Histological type (GX/G1/G2/G3)	44/4/27/4	11/5/20/3	0.01
Lymphatic invasion	3	5	0.07
Microvascular invasion	18	9	0.42
Perineural invasion	3	0	0.22
UICC T1/T2/T3/T4	13/42/19/1	10/16/10/1	0.76
UICC N0/N1/N2	76/3/0	39/0/0	0.22
Largest tumour diameter	48 (48.4/17–220)	52 (60/30–220)	0.07
Number of tumours	2.5 (4.2/1–25)	3 (4/1–20)	0.84
Postoperative course			
Overall survival in d	1964	1313	0.06
1 y survival (%)	75.9	84.2	0.85
5 y survival (%)	56.4	40	0.84
Disease-free survival in d	2705	1604	0.006
Recurrence (%)	27.8	38.5	0.25
Initial recurrence site			
Peritoneum	6	3	0.96
Local (Liver)	10	6	0.71
Lung	12	10	0.22
Lymph node	5	5	0.27
Bones	5	6	0.14
Adrenal gland	4	2	0.96

CPT, Child-Pugh-Turcotte Score for cirrhosis mortality; AFP, alpha-fetoprotein; GX, grade cannot be assessed due to neoadjuvant therapy; UICC, Union for International Cancer Control.

**Table 3:** Cox proportional hazard analysis of risk factors for disease-free survival; AFP, alpha-fetoprotein; Complication, complication of Clavien-Dindo ≥ 3a.

Factors	Disease-Free Survival			
	Univariate		Multivariate	
	HR (95% CI)	*p* Value	HR (95% CI)	*p* Value
Male (vs. female)	1.12 (0.45–2.57)	0.744		
Complication (grade ≥ 3)	2.10 (0.30–15.3)	0.371		
Largest tumour diameter > 50 mm (vs. <50 mm)	0.79 (0.40–1.89)	0.492		
>3 tumours (vs. <3 tumours)	0.81 (0.33–1.39)	0.566		
Tumour differentiation G1 or G2 (vs. G3)	6.34 (2.51–17.2)	0.0002	4.47 (0.99–19.1)	0.0324
Microvascular invasion (V1 vs. V0)	8.21 (3.11–17.85)	<0.0001	1.89 (1.12–6.47)	0.169
The N1, 2 (vs. N0)	3.39 (1.41–8.8)	0.08		
Lymphatic permeation	1.84 (0.54–3.23)	0.226		
Bridging (vs. no bridging)	7.50 (3.46–12.44)	0.0001	2.67 (1.86–6.11)	0.01
Downstaging (vs. no downstaging)	2.21 (0.76–4.21)	0.652		
AFP > 1000 ng/mL (vs. <1000 ng/mL)	4.19 (0.24–14.73)	0.09		
labMELD > 20 (vs. <20)	1.43 (0.73–4.10)	0.429		
Preoperative ICU-stay (vs. no hospitality)	2.13 (0.47–3.56)	0.584		
Waiting time > 1 y (vs. <1 y)	2.42 (0.75–4.83)	0.08		
LDLT (vs. LT)	1.28 (0.55–2.61)	0.12		

**Table 4:** Comparison of HCC classification systems.

	MILAN		UCSF		“5-5-500”-rule		BCLC			Toronto		“Up-to-Seven”	
	Inside Milan	Outside Milan	Inside UCSF	Outside UCSF	Inside 5-5- 500	Outside 5-5-500	BCLC A	BCLC B	BCLC C	Inside Toronto	Outside Toronto	Inside UTSC	Outside UTSC
Total (n =)	125	120	47	73	40	80	25	64	31	104	16	45	75
OS in d	1979.9	1748.8	1646.5	1798.75	1304.7	1958.2	1880.8	1744.5	1618.7	1776	1508.9	1524.9	1869.7
5 y survival (%)	79.9%	51%	60.9%	46.4%	53%	50.8%	64%	52.4%	40%	54.4%	33.3%	63.2%	47.5%
1 y survival (%)	99.5%	78.64%	88.4%	76.1%	79%	80.8%	88%	82%	70%	80.4%	73.3%	90.5%	75.8%
DFS in d	2951.5	2341.1	2098.3	2504.5	1784.9	2618.2	2639.2	2432.2	1909.9	2464	1537.1	1992.8	2549
recurrence (%)	11.2%	31.3%	19.1%	37.5%	12.5%	40%	12%	29.7%	48.4%	26%	62.5%	13.3%	41.3%
LT (n =)	106	79	33	46	24	55	13	46	20	70	9	30	49
LDLT (n =)	19	39	14	27	16	25	12	18	11	34	7	15	26

## Data Availability

The data presented in this study are available on request from the corresponding author.

## References

[1] Da B.L., Suchman K.I., Lau L., Rabiee A., He A.R., Shetty K., Yu H., Wong L.L., Amdur R.L., Crawford J.M. (2022). Pathogenesis to Management of Hepatocellular Carcinoma. Genes Cancer.

[2] Dopazo C., Søreide K., Rangelova E., Mieog S., Carrion-Alvarez L., Diaz-Nieto R., Primavesi F., Stättner S. (2024). Hepatocellular Carcinoma. Eur. J. Surg. Oncol..

[3] Mazzaferro V., Regalia E., Doci R., Andreola S., Pulvirenti A., Bozzetti F., Montalto F., Ammatuna M., Morabito A., Gennari L. (1996). Liver Transplantation for the Treatment of Small Hepatocellular Carcinomas in Patients with Cirrhosis. N. Engl. J. Med..

[4] Mazzaferro V., Llovet J.M., Miceli R., Bhoori S., Schiavo M., Mariani L., Camerini T., Roayaie S., Schwartz M.E., Grazi G.L. (2009). Predicting Survival after Liver Transplantation in Patients with Hepatocellular Carcinoma beyond the Milan Criteria: A Retrospective, Exploratory Analysis. Lancet Oncol..

[5] Shehta A., Lee J.-M., Suh K.-S., Kim H.-C., Hong S.K., Cho J.-H., Yi N.-J., Lee K.-W. (2020). Bridging and Downstaging Role of Trans-Arterial Radio-Embolization for Expected Small Remnant Volume before Liver Resection for Hepatocellular Carcinoma. Ann. Hepato-Biliary-Pancreat. Surg..

[6] Kim M., Hui K.M., Shi M., Reau N., Aloman C. (2022). Differential Expression of Hepatic Cancer Stemness and Hypoxia Markers in Residual Cancer after Locoregional Therapies for Hepatocellular Carcinoma. Hepatol. Commun..

[7] Ettorre G.M., Laurenzi A. (2019). Other “bridge” therapies for liver transplantation: RFA, TACE, and TARE. Liver Transplantation and Hepatobiliary Surgery, Interplay of Technical and Theoretical Aspects.

[8] Gao Q., Anwar I.J., Abraham N., Barbas A.S. (2021). Liver Transplantation for Hepatocellular Carcinoma after Downstaging or Bridging Therapy with Immune Checkpoint Inhibitors. Cancers.

[9] Zori A.G., Ismael M.N., Limaye A.R., Firpi R., Morelli G., Soldevila-Pico C., Suman A., Vogel J.D., Lazarowicz M., Geller B.S. (2020). Locoregional Therapy Protocols With and Without Radioembolization for Hepatocellular Carcinoma as Bridge to Liver Transplantation. Am. J. Clin. Oncol..

[10] Makary M.S., Bozer J., Miller E.D., Diaz D.A., Rikabi A. Long-Term Clinical Outcomes of Yttrium-90 Transarterial Radioembolization for Hepatocellular Carcinoma: A 5-Year Institutional Experience. Acad. Radiol..

[11] Zhang J., Hu C., Xie X., Qi L., Li C., Li S. (2023). Immune Checkpoint Inhibitors in HBV-Caused Hepatocellular Carcinoma Therapy. Vaccines.

[12] Wassmer C.-H., Hajji S.E., Papazarkadas X., Compagnon P., Tabrizian P., Lacotte S., Toso C. (2023). Immunotherapy and Liver Transplantation: A Narrative Review of Basic and Clinical Data. Cancers.

[13] Wehrenberg-Klee E., Goyal L., Dugan M., Zhu A.X., Ganguli S. (2018). Y-90 Radioembolization Combined with a PD-1 Inhibitor for Advanced Hepatocellular Carcinoma. Cardiovasc. Interv. Radiol..

[14] Yao F.Y., Xiao L., Bass N.M., Kerlan R., Ascher N.L., Roberts J.P. (2007). Liver Transplantation for Hepatocellular Carcinoma: Validation of the UCSF-Expanded Criteria Based on Preoperative Imaging. Am. J. Transplant..

[15] Sapisochin G., Goldaracena N., Laurence J.M., Dib M., Barbas A., Ghanekar A., Cleary S.P., Lilly L., Cattral M.S., Marquez M. (2016). The Extended Toronto Criteria for Liver Transplantation in Patients with Hepatocellular Carcinoma: A Prospective Validation Study. Hepatology.

[16] Takishima T., Haruki K., Taniai T., Furukawa K., Horiuchi T., Onda S., Yanagaki M., Shirai Y., Hamura R., Ikegami T. (2023). The Japanese 5-5-500 Rule Predicts Prognosis of Hepatocellular Carcinoma After Hepatic Resection. Anticancer Res..

[17] Reig M., Forner A., Rimola J., Ferrer-Fàbrega J., Burrel M., Garcia-Criado Á., Kelley R.K., Galle P.R., Mazzaferro V., Salem R. (2022). BCLC Strategy for Prognosis Prediction and Treatment Recommendation: The 2022 Update. J. Hepatol..

[18] Lubel J.S., Roberts S.K., Strasser S.I., Shackel N. (2021). Australian Recommendations for the Management of Hepatocellular Carcinoma. Med. J. Aust..

[19] Seehofer D., Petrowsky H., Schneeberger S., Vibert E., Ricke J., Sapisochin G., Nault J.-C., Berg T. (2022). Patient Selection for Downstaging of Hepatocellular Carcinoma Prior to Liver Transplantation—Adjusting the Odds?. Transpl. Int..

[20] DSO Jahresbericht der Deutschen Stiftung für Organspende 2021. https://dso.de/BerichteTransplantationszentren/Grafiken%20D%202021%20Leber.pdf.

[21] Goldaracena N., Gorgen A., Doyle A., Hansen B.E., Tomiyama K., Zhang W., Ghanekar A., Lilly L., Cattral M., Galvin Z. (2019). Live Donor Liver Transplantation for Patients with Hepatocellular Carcinoma Offers Increased Survival vs. Deceased Donation. J. Hepatol..

[22] Nadalin S., Capobianco I., Panaro F., Francesco F.D., Troisi R., Sainz-Barriga M., Muiesan P., Königsrainer A., Testa G. (2016). Living Donor Liver Transplantation in Europe. Hepatobiliary Surg. Nutr..

[23] Malinchoc M., Kamath P.S., Gordon F.D., Peine C.J., Rank J., Borg P.C.J. (2000). ter. A Model to Predict Poor Survival in Patients Undergoing Transjugular Intrahepatic Portosystemic Shunts. Hepatology.

[24] Lencioni R., Llovet J. (2010). Modified RECIST (MRECIST) Assessment for Hepatocellular Carcinoma. Semin. Liver Dis..

[25] Eisenhauer E.A., Therasse P., Bogaerts J., Schwartz L.H., Sargent D., Ford R., Dancey J., Arbuck S., Gwyther S., Mooney M. (2009). New Response Evaluation Criteria in Solid Tumours: Revised RECIST Guideline (Version 1.1). Eur. J. Cancer.

[26] Vogel A., Meyer T., Sapisochin G., Salem R., Saborowski A. (2022). Hepatocellular Carcinoma. Lancet.

[27] Hameed B., Mehta N., Sapisochin G., Roberts J.P., Yao F.Y. (2014). Alpha-fetoprotein Level > 1000 Ng/ML as an Exclusion Criterion for Liver Transplantation in Patients with Hepatocellular Carcinoma Meeting the Milan Criteria. Liver Transplant..

[28] Feng L.-H., Zhu Y.-Y., Zhou J.-M., Wang M., Wang L., Xu W.-Q., Zhang T., Mao A.-R., Cong W.-M., Dong H. (2023). A Practical Risk Classification of Early Recurrence in Hepatocellular Carcinoma Patients with Microvascular Invasion after Hepatectomy: A Decision Tree Analysis. Ann. Surg. Oncol..

[29] Toso C., Meeberg G., Hernandez-Alejandro R., Dufour J., Marotta P., Majno P., Kneteman N.M. (2015). Total Tumor Volume and Alpha-fetoprotein for Selection of Transplant Candidates with Hepatocellular Carcinoma: A Prospective Validation. Hepatology.

[30] Bhangui P., Vibert E., Majno P., Salloum C., Andreani P., Zocrato J., Ichai P., Saliba F., Adam R., Castaing D. (2011). Intention-to-treat Analysis of Liver Transplantation for Hepatocellular Carcinoma: Living versus Deceased Donor Transplantation. Hepatology.

[31] Yao F.Y., Ferrell L., Bass N.M., Watson J.J., Bacchetti P., Venook A., Ascher N.L., Roberts J.P. (2001). Liver Transplantation for Hepatocellular Carcinoma: Expansion of the Tumor Size Limits Does Not Adversely Impact Survival. Hepatology.

[32] Sotiropoulos G.C., Lang H., Nadalin S., Neuhäuser M., Molmenti E.P., Baba H.A., Paul A., Saner F.H., Weber F., Hilgard P. (2007). Liver Transplantation for Hepatocellular Carcinoma: University Hospital Essen Experience and Metaanalysis of Prognostic Factors. J. Am. Coll. Surg..

[33] Vakili K., Pomposelli J.J., Cheah Y.L., Akoad M., Lewis W.D., Khettry U., Gordon F., Khwaja K., Jenkins R., Pomfret E.A. (2009). Living Donor Liver Transplantation for Hepatocellular Carcinoma: Increased Recurrence but Improved Survival. Liver Transplant..

[34] Sandro S.D., Slim A.O., Giacomoni A., Lauterio A., Mangoni I., Aseni P., Pirotta V., Aldumour A., Mihaylov P., Carlis L.D. (2009). Living Donor Liver Transplantation for Hepatocellular Carcinoma: Long-Term Results Compared With Deceased Donor Liver Transplantation. Transplant. Proc..

[35] Sandhu L., Sandroussi C., Guba M., Selzner M., Ghanekar A., Cattral M.S., McGilvray I.D., Levy G., Greig P.D., Renner E.L. (2012). Living Donor Liver Transplantation versus Deceased Donor Liver Transplantation for Hepatocellular Carcinoma: Comparable Survival and Recurrence. Liver Transplant..

[36] Park M.-S., Lee K.-W., Suh S.-W., You T., Choi Y., Kim H., Hong G., Yi N.-J., Kwon C.-H.D., Joh J.-W. (2014). Living-Donor Liver Transplantation Associated With Higher Incidence of Hepatocellular Carcinoma Recurrence Than Deceased-Donor Liver Transplantation. Transplant. J..

[37] Hwang S., Lee S., Joh J., Suh K., Kim D. (2005). Liver Transplantation for Adult Patients with Hepatocellular Carcinoma in Korea: Comparison between Cadaveric Donor and Living Donor Liver Transplantations. Liver Transplant..

[38] Sandri G.B.L., Rayar M., Qi X., Lucatelli P. (2018). Liver Transplant for Patients Outside Milan Criteria. Transl. Gastroenterol. Hepatol..

[39] Ramos F., Castellanos M., las Heras N.D., Escalante F., Fernandez-Ferrero S., Vidal M.J., Villalobos M.L. (2020). ECOG Performance Status Shows a Stronger Association with Treatment Tolerability Than Some Multidimensional Scales in Elderly Patients Diagnosed with Hematological Malignancies. Blood.

[40] Martino M.D., Lai Q., Lucatelli P., Damato E., Calabrese A., Masci G.M., Parisse S., Sedati P., Merli M., Mennini G. (2021). Comparison of Up-to-Seven Criteria with Milan Criteria for Liver Transplantation in Patients with HCC. Trends Transplant..

[41] Rauchfuß F., Dondorf F., Fahrner R., Tautenhahn H.-M., Ardelt M., Settmacher U. (2017). Searching the Ideal Hepatocellular Carcinoma Patient for Liver Transplantation: Are the Toronto Criteria a Step in the Right Direction?. Hepatobiliary Surg. Nutr..

[42] Zheng S.-S., Xu X., Wu J., Chen J., Wang W.-L., Zhang M., Liang T.-B., Wu L.-M. (2008). Liver Transplantation for Hepatocellular Carcinoma: Hangzhou Experiences. Transplantation.

[43] Yan P., Yan L.-N. (2003). Staging of Hepatocellular Carcinoma. Hepatobiliary Pancreat. Dis. Int. HBPD INT.

[44] Yap A.Q., Chen C.-L., Yong C.-C., Kuo F.-Y., Wang S.-H., Lin C.-C., Liu Y.-W., Lin T.-L., Li W.-F., Millan C.A. (2013). Clinicopathological Factors Impact the Survival Outcome Following the Resection of Combined Hepatocellular Carcinoma and Cholangiocarcinoma. Surg. Oncol..

[45] Bhatti A.B.H., Naqvi W., Khan N.Y., Zia H.H., Dar F.S., Khan Z.A., Rana A. (2022). Living Donor Liver Transplantation for Advanced Hepatocellular Carcinoma Including Macrovascular Invasion. J. Cancer Res. Clin. Oncol..

[46] Lin C.-C., Chen C.-L. (2016). Living Donor Liver Transplantation for Hepatocellular Carcinoma Achieves Better Outcomes. Hepatobiliary Surg. Nutr..

[47] Bhatti A.B.H., Waheed A., Khan N.A. (2021). Living Donor Liver Transplantation for Hepatocellular Carcinoma: Appraisal of the United Network for Organ Sharing Modified TNM Staging. Front. Surg..

[48] Wong T.C.L., Ng K.K.C., Fung J.Y.Y., Chan A.A.C., Cheung T.-T., Chok K.S.H., Dai J.W.C., Lo C.-M. (2019). Long-Term Survival Outcome Between Living Donor and Deceased Donor Liver Transplant for Hepatocellular Carcinoma: Intention-to-Treat and Propensity Score Matching Analyses. Ann. Surg. Oncol..

[49] Lei J.Y., Wang W.T., Yan L.N. (2014). Hangzhou Criteria for Liver Transplantation in Hepatocellular Carcinoma: A Single-Center Experience. Eur. J. Gastroenterol. Hepatol..

[50] Ivanics T., Claasen M.P., Samstein B., Emond J.C., Fox A.N., Pomfret E., Pomposelli J., Tabrizian P., Florman S.S., Mehta N. (2023). Living Donor Liver Transplantation (LDLT) for Hepatocellular Carcinoma (HCC) within and Outside Traditional Selection Criteria: A Multicentric North American Experience. Ann. Surg..

[51] Chen J., Xu X., Ling Q., Wu J., Zheng S. (2007). Role of Pittsburgh Modified TNM Criteria in Prognosis Prediction of Liver Transplantation for Hepatocellular Carcinoma. Chin. Med. J..

[52] Pons F., Varela M., Llovet J.M. (2005). Staging Systems in Hepatocellular Carcinoma. HPB.

